# A comprehensive review on pathogenesis, associations, clinical findings, and treatment of livedoid vasculopathy

**DOI:** 10.3389/fmed.2022.993515

**Published:** 2022-12-08

**Authors:** Mireia Seguí, Mar Llamas-Velasco

**Affiliations:** Department of Dermatology, Hospital Universitario de la Princesa, Madrid, Spain

**Keywords:** livedoid vasculopathy (LV), atrophie blanche, livedo racemosa, retiform purpura, thrombosis, rivaroxaban, anticoagulant, antiplatelet

## Abstract

Livedoid vasculopathy (LV) is a thrombo-occlusive vasculopathy that involves the dermal vessels. Clinically, it is characterized by the presence of painful purpuric ulcers on the lower extremities. Histopathologically, it shows intraluminal fibrin deposition and thrombosis, segmental hyalinization, and endothelial proliferation. It is important to notice that the term “atrophie blanche” is descriptive and it includes not only patients with LV but also patients with a combination of vasculitis and vasculopathy, that is, LV and medium-sized vasculitis such as cutaneous polyarteritis nodosa (PANc). Diagnosis is based on a proper clinicopathological correlation, excluding the main differential diagnosis and considering vasculitis as a mimicker or concomitant diagnosis. Coagulation disorders must also be studied although they are not found in all LV. Its frequency is reviewed as well. Treatment of LV is challenging, and different therapies have been attempted. Among them, pain management, wound care, control of cardiovascular risk factors, and both antiplatelets and anticoagulants, mostly rivaroxaban, are the main therapies used. These different therapies as well as their degree of evidence are reviewed.

## Introduction

Livedoid vasculopathy (LV), first described by Milian (1), is a thrombo-occlusive vasculopathy involving the postcapillary venules of the dermis. It can be considered a syndromic concept, including patients with a locoregional manifestation of a venous thrombus guided by three key factors: (1) flow disruption, (2) endothelial injury, and (3) coagulation disorder (2). Different names, including *atrophie blanche*, segmental hyalinizing vasculitis, Milian white atrophy, livedo reticularis with summer ulceration, livedo vasculitis, segmental hyalinizing vasculitis, or painful purpuric ulcers with a reticular pattern of the lower extremities (PURPLE), have also been used to refer to patients with LV. Bard and Winkelman were the first authors to use the term LV in 1967 (3).

## Epidemiology

Livedoid vasculopathy is a rare disease, with an estimated incidence rate of 1 in 100,000 (4). This disease predominantly affects women, with a female-to-male ratio of 3:1, and it occurs mainly in young to middle-aged patients. As reported in the literature, the median age ranges from 35 to 53 years (5, 6). It is noteworthy that there is a 5-year delay from the first symptoms until an accurate diagnosis and thus promoting a better knowledge of LV may help decrease the diagnostic delay (7).

## Etiopathogenesis

Livedoid vasculopathy can be classified as primary (idiopathic) and secondary when coagulation disorders are associated (4, 8). Although the pathogenesis of LV remains unclear, it is thought to involve a locally favored alteration in either an increased local or systemic thrombotic activity or a decreased fibrinolytic alteration, that is a coagulation disturbance, that leads to the formation of fibrin thrombi within superficial dermal blood vessels (9). The resulting tissue hypoxia within the involved area of the dermis leads to poor wound healing and an ineffective barrier, thus enhancing the risk of infection (10).

Livedoid vasculopathy may appear to be associated with any conditions related to stasis, autoimmune connective tissue diseases, thrombophilias, or neoplasms (4).

On the one hand, due to the clinical evidence of increased ambient temperature as a trigger, this disease raised the possible existence of “pyroglobulins” analogous to cryoglobulins; however, this was not evidenced and was just a hypothetical way of explaining why we have cases with no clear reason to explain the coagulation alteration (11).

On the other hand, a large number of hypercoagulable states have been associated with LV, including antiphospholipid antibodies, factor V Leiden mutation, protein C and S deficiency, prothrombin mutation, antithrombin III deficiency, hyperhomocysteinemia, and increased levels of lipoprotein(a) (4, 8, 12). The fact that there is a good response to treatment with anticoagulants, fibrinolytics, and antiplatelet drugs supports the suggested underlying prothrombotic pathogenesis (4).

However, the underlying coagulation disorders are diverse and have been found variably in the literature. In a retrospective study of 75 Brazilian patients with LV, about 66% of the cases had thrombophilic factors, with lipoprotein(a) being the most common thrombophilic factor detected in 30 (41.66%) of 72 patients (13). In another recent study, prothrombotic parameters were found in 11 (44%) of 25 patients with LV (14). Increased homocysteine in 10 of 12 patients (83%) and lipoprotein(a) in 5 of 12 patients 42%) were the most frequently observed. Few authors have investigated Lp(a) levels in patients with LV, not being included in the rest of the studies. In a prospective study of 34 patients, 18 of them (52%) presented laboratory abnormalities of procoagulant conditions (15). The most common prothrombotic factors observed were antiphospholipid antibodies (17.64%), factor V Leiden mutation in heterozygosis (17.64%), and protein C and/or S deficiency (8.82%). In another study, 29 patients were tested for abnormalities in coagulation, 12 of them (41.4%) were found positive, with the anticardiolipin antibody being the most frequent (16). Table 1 provides data about the thrombophilic findings in articles with more than 30 cases reported.

**TABLE 1:** Thrombophilic findings in livedoid vasculopathy case series.

	Criado et al. (13)	Di Giacomo et al. (15)	Hairston et al. (16)
Thrombophilic factors	48/72 (66.66%)	18/34 (52%)	12/29 (41.4%)
Factor V (Leiden) mutation (G1691A)	3	6	2
Prothrombin gene mutation (G20210A)	2	1	1
Protein C	2	3	2
Protein S	3		
Antithrombin III	3	1	–
Lipoprotein (a)	30	–	–
Factor VIII	9	–	–
Factor IX	5	–	–
Homocystein	5	2	3
Lupus anticoagulant	7	1	5
IgM anticardiolipin antibodies	10	4	5
IgG anticardiolipin antibodies	10	0	1
IgM + IgG anticardiolipin antibodies	8	2	2

Other genetic disorders associated with the pathogenesis of LV are the presence of polymorphisms in plasminogen activator inhibitor-1 (PAI-1) and methylenetetrahydrofolate reductase (MTHFR). A recent systematic review of genetic variants in LV found that PAI-1 675 4G/5G was the most common, accounting for 85.26% of the patients, followed by PAI-1 A844G, MTHFR C677T, and MTHFR A1298C variants (17). It is suggested that the distribution of variants may be related to geographical location or ethnicity. Prothrombin G20210A and factor V G1691A were mostly seen in patients with LV from Europe, North America, and South America.

Regarding the association of clinical phenotypes and certain thrombophilic factors or genetic variants, as discussed, no genetic or thrombophilic factors have been associated with a particular clinical picture. Therefore, we cannot assume that LV is primary or secondary based on the clinical or histopathological findings.

Livedoid vasculopathy is usually limited to the lower legs, thus it is assumed that local factors such as stasis and temperature are important factors related to its pathogenesis. Furthermore, additional unknown individual trigger factors must also play a role since only a small number of patients suffering from different coagulopathies develop LV (18, 19).

## Clinical features

Livedoid vasculopathy is a chronic disease with periodic and recurrent exacerbations. Clinically, it is characterized by three main typical features: livedo racemosa, skin ulcerations, and atrophie blanche (18, 19). The disease is mostly bilateral (4).

**FIGURE 1:**
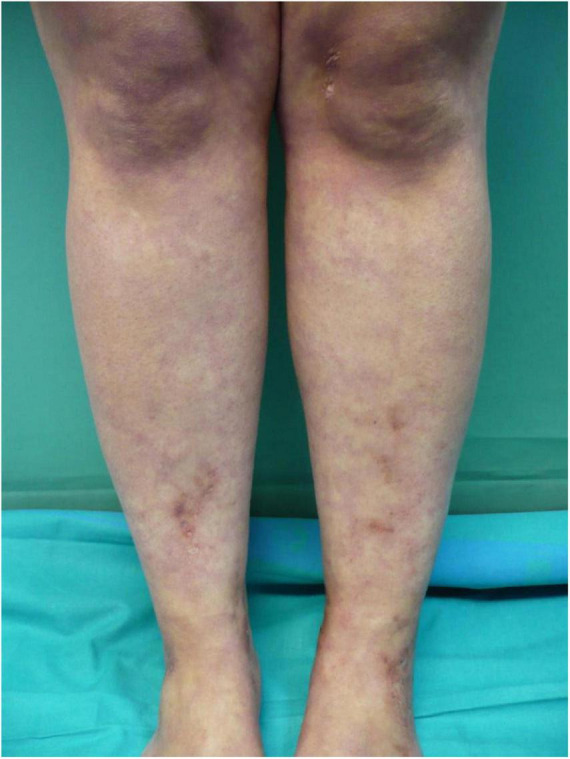
Livedo racemosa lesions on the legs with erythematous to violaceous broken network pattern.

Livedo racemosa is defined as a persistent, erythematous to violaceous discoloration of the skin characterized by broken, branched, discontinuous, and irregular pattern (14) (Figure 1). Livedo racemosa is frequently and consistently associated with LV, but it is not a specific feature as it can appear also in a wide range of occlusive vasculopathies (9). In a recent study, Weishaupt et al. found that livedo racemosa was present in 85% of patients with LV (14). It usually affects the lower limbs but can also affect the upper limbs or the trunk when associated with LV. Livedo racemosa may be viewed as an early manifestation of LV (9).

**FIGURE 2:**
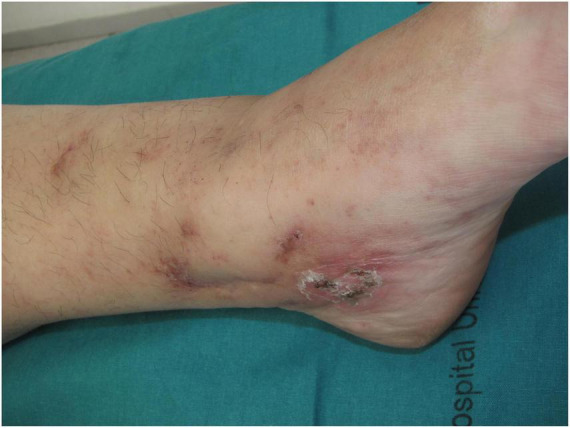
Retiform purpura with small crusted ulcers distributed on the ankles.

**FIGURE 3:**
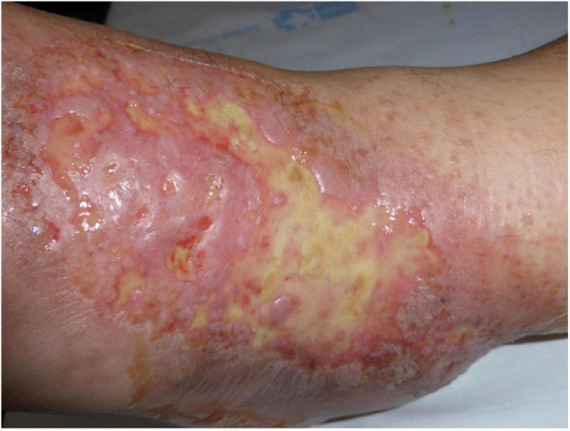
Active ulcers on the ventral distal lower leg with associated edema.

Other clinical features of LV include purpuric macules, papules, and retiform purpura, followed by the formation of acute-onset, painful, small crusted ulcers (Figure 2). Ulcers represent the active stage of the disease (9). They are usually located beneath the knees, the most compromised location being the ankle area (medial more common than lateral), followed by the dorsal foot and the ventral distal lower leg (14) (Figure 3). Some authors have also found lesions on the upper extremities in a small proportion of patients (20). These ulcers are typically small (<1 cm), painful, with a punched-out appearance, frequently bilateral, and recurrent. Edema can also be present. Burning pain, sometimes excruciating, often precedes the ulceration and may be a prodromal clue for this diagnosis (10). A cross-sectional study showed that patients with LV have significantly impaired quality of life, especially during disease activity, having an impact on their psychological, physical, and social aspects of life (21).

**FIGURE 4:**
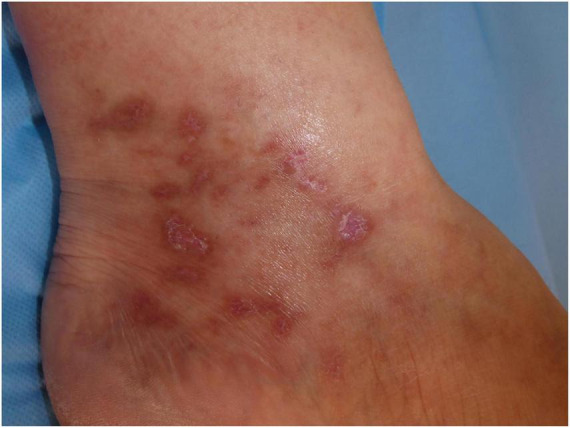
Atrophie blanche with small, round scars surrounded by hyperpigmentation and telangiectasias.

The ulcerated lesions slowly tend to heal within 3–4 months resulting in the so-called “*atrophie blanche*,” stellate porcelain white atrophic scars surrounded by hyperpigmentation and telangiectasias (10) (Figure 4). *Atrophie blanche* is the residual state of ulcers of LV; therefore, it is located where these appear (14). *Atrophie blanche*, also known as *capillaritis alba*, may also be seen in many other conditions such as chronic venous insufficiency or some autoimmune connective tissue diseases (lupus erythematosus, dermatomyositis), and even associated with medium-sized vessel vasculitis (8, 22, 23).

**FIGURE 5:**
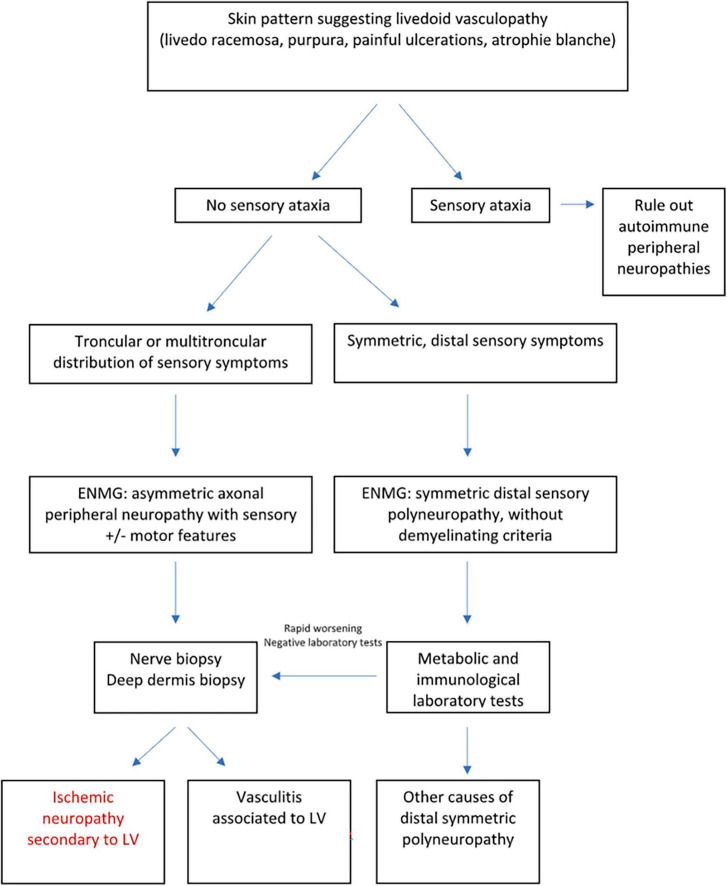
Diagnostic approach of peripheral neuropathies associated with LV adapted from Soulages et al. (24).

Peripheral neuropathy is the only known extracutaneous manifestation of LV. Although rarely described in the literature, a recent study has revealed a higher incidence of peripheral neuropathy (50% of patients), including cases of mononeuritis multiplex, sensory polyneuropathy, and small fiber neuropathy (5). The main etiopathogenic explanation is based on the occurrence of thrombotic disease involving vessels of the nerve and thus causing nerve injury due to hypoxia (24). In a study involving 16 patients with peripheral neuropathy and LV, asymmetric axonal polyneuropathy was found as the most frequent EMG pattern, followed by sensorimotor mononeuropathy in one case. The most frequently affected sensory nerve was the sural nerve, followed by the superficial fibular, median and ulnar nerves [four cases each (24)]. Peripheral neuropathy in LV requires further investigation because the conventional techniques (EMG, nerve biopsy) explore only large nerve fibers, which is in contrast to our study which involved smaller nerves (Figure 5). Peripheral neuropathy is probably underestimated and would explain the high percentage of patients with neuropathic pain persisting after the healing of ulcers, despite having a normal EMG (5).

Dermoscopic features of LV consist of pink or white background, irregular linear and glomerular vessels, central crusted ulcers, and ivory-white areas associated with peripheral pigmentation in a reticular pattern (25, 26).

On histopathological examination, the ivory white areas correlate with dermal fibrosis, the reticular pigmentation corresponds to epidermal basal layer hyperpigmentation or melanin within melanophages in the dermal papillae, and the vascular structures correlate with dilated vessels and proliferation of capillaries (25).

## Histopathological features

**FIGURE 6:**
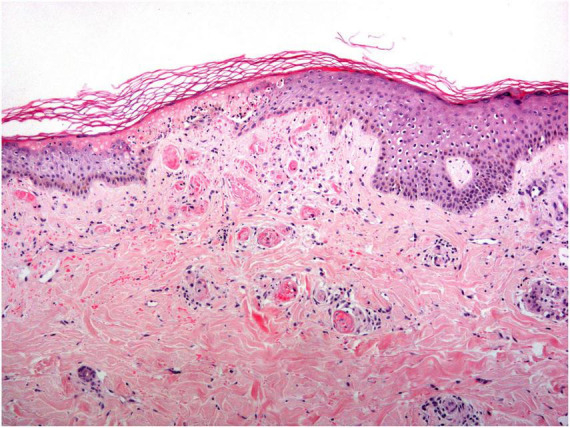
Livedoid vasculopathy histology: An acutely necrotic epidermis is observed overlying an area where all the vessels in the papillary dermis are completely occluded by a hyaline thrombi.

The histopathological findings of LV are characterized by occlusion of dermal blood vessels due to intraluminal fibrin deposition and thrombosis, segmental hyalinization, and endothelial proliferation (8) (Figure 6). No signs of true vasculitis are found as there are no neutrophilic polymorphonuclear leukocytes permeating the vessel wall or surrounding the dermal vessels (27).

Histopathological changes depend on the stage of the lesion. In the early stage, the hyaline thrombus is formed in the lumen of small vessels in the mid and papillary dermis, and it is sometimes associated with the deposition of fibrinoid material on the vessel walls and in the perivascular estroma (4, 8). In addition to these angiocentric findings, most cases present with overlying ulceration (infarction) of the epidermis and adjacent superficial dermis. A sparse perivascular lymphocytic infiltrate may be seen, with no signs of leukocytoclastic vasculitis. Extravasation of red blood cells in the superficial dermis can be found as well. A non-specific papillary dermis increase in small blood vessels is also common (4).

Partially developed lesions show a hyalinization and thickening of the vessel walls in the papillary dermis, followed by secondary endothelial proliferation (8).

Fully developed lesions demonstrate dermal sclerosis and scarring with some dilated lymphatic and epidermal atrophy (4).

Direct immunofluorescence, when done in patients with LV, demonstrates deposition of immunoglobulins, complement, and fibrin (8, 28). Direct immunofluorescence (IFD) study in patients with LV showed positive immunoreactants ranging from 42.9 to 100% (28). C3 and IgM are the most common immunoreactants found, followed by IgA and IgG. The most commonly reported IFD pattern is immunoreactant deposition in blood vessels and at the dermoepidermal junction. Nuttawong et al. reported that older patients and those with more recent lesions (<6 months) have a significantly higher percentage of positive IFD results for LV than younger patients and those with older lesions (≥6 months) (28).

## Diagnosis

The diagnostic criteria of LV are not well-defined (14). This could be due to limited or missing data referred to this entity that will need clarification through clinicopathological studies to encompass that diverse range of laboratory findings. Clinical, histopathological, and laboratory data are necessary to make a correct diagnosis of LV (4).

There is no list of diagnostic criteria, but LV must be suspected in patients with recurrent small painful ulcerations mainly around the ankles when the temperature rises. Edema can be present and irregular lesions of atrophie blanche may be found either in previously ulcerated skin or without ulceration. Livedo racemosa as well as papules or dark irregular purpura that evolve to ulceration can be found along with the previously described features (11).

When a clinical suspicion of LV exists, a skin biopsy is required to confirm and rule out other differential diagnoses. The biopsy specimen should be taken of the immediate borders of a new ulcer and include both healthy surrounding skin and the eventual ulcer (9). It should ideally be an incisional biopsy containing subcutaneous fat.

Once the presence of LV is confirmed, an accurate laboratory evaluation must be carried out to exclude any possible underlying diseases (Table 2). Regarding prothrombotic markers, nowadays, the value of testing them for a particular patient can be discussed as they do not always change the therapeutic approach and no cost-effectiveness studies have been made. Alavi and Kerk also challenged the idea of testing all patients (4, 19). In any case, if we want to analyze in-depth pathogenesis, this type of analysis would be helpful. Detailed laboratory investigations for connective tissue diseases are also recommended. In addition, it should also be ruled out the presence of paraproteinemia and underlying infections (4).

**TABLE 2:** Laboratory testing for livedoid vasculopathy.

Disease	Investigation
Hypercoagulable states	Tests of Haemostasis: PT, aPTT, Fibrinogen, D-dimer Factor V Leiden mutation Prothrombin G20210A mutation Antithrombin III deficiency Protein C and S deficiency Homocystein Folic acid, vitamin B12 and vitamin B6 Lipoprotein (a) Methylene-tetrahydrofolate-reductase C677T mutation Plasminogen activator inhibitor Anticardiolipin antibodies (IgM and IgG) Lupus anticoagulant Anti-b2-glioprotein I antibodies Cryoglobulin Cold agglutinins
Connective tissue diseases	ANA ANCA ENA Anti-Ro Anti-La Anti-CCP Rheumatoid factor Complement (C3, C4)
Paraproteinemias	Cryofibrinogen Immunoglobulin, Kappa and lambda chain Protein electrophoresis, immunofixation
Infections	Hepatitis B and C HIV

Other appropriate tests for the diagnostic investigation of LV include venous and artery Doppler ultrasound, pulse examination, and ankle-brachial index, to study venous insufficiency and arterial peripheral disease. It is also advisable to rule out a pregnancy.

Although not located in the lower legs, SARS-CoV2 infection is related to the presence of a thrombotic occlusive vasculopathy in the skin (29). With the previous knowledge, as SARS-CoV2 presents endothelial tropism and has the ability to favor microthrombosis in different tissues, it is not unexpected that the viral infection can worsen LV in previously affected patients, even in non-severe cases (30).

Despite performing a deep investigation into the underlying conditions, 20% of all the cases are classified as idiopathic LV (9).

## Differential diagnosis

It includes many diseases where ulcers, white stellate atrophy, or pain involves mostly the legs. Most cases of chronic venous insufficiency can be identified by the presence of stasis dermatitis along with varicose veins and an abnormal venous Doppler.

Regarding peripheral arterial disease, the presence of cardiovascular risk factors, intermittent claudication, and abnormal arterial Doppler along with an altered ankle-brachial index test leads us to rule out this diagnosis.

There are many vasculitis where inflammatory retiform purpura may appear as they involve both small and median-size vessels. ANCA-vasculitis and IgA vasculitis are within this group of diseases. A skin biopsy will show a real vasculitis, while direct IF will show IgA and, to a lesser extent, IgM or IgG. In ANCA-associated vasculitis, p-ANCA or c-ANCA is positive and other organ damage such as renal, pulmonary, or neurological diseases also occur. Antiphospholipid antibody syndrome may produce stellate scarring in the lower limb, but the diagnosis requires clinical and laboratory criteria based on the International Consensus Statement (31).

Regarding cutaneous arteritis, previously named cutaneous polyarteritis nodosa, although livedo, nodules, and mononeuritis multiplex are found, a skin biopsy will show a medium-sized artery involvement rather than the histopathological findings of LV.

Arteritis macular is a lymphocytic arteritis of dermo-hypodermal vessels characterized by a fibrin hyalinized ring and hyperpigmented or pink macules (32). Some authors have considered this entity as a latent form of cutaneous polyarteritis nodosa (33, 34). Typical histopathology and, most commonly, the absence of ulceration and scarring are useful to rule out this entity.

As previously stated, it is noteworthy to highlight the finding of a coexistence of cutaneous panarteritis and LV (35) as well as some cases with other underlying subcutaneous necrotizing vasculitis (36).

Degos disease presents clinically characteristic porcelain white atrophic lesions surrounded by dilated vessels, but any area of the body can be involved and all the lesions are similar in size.

Regarding Sneddon syndrome, livedo racemosa is also found in LV. But this group of patients is characterized by the presence of cerebrovascular stroke and an underlying mutation in CERC1 (37).

## Treatment

**FIGURE 7:**
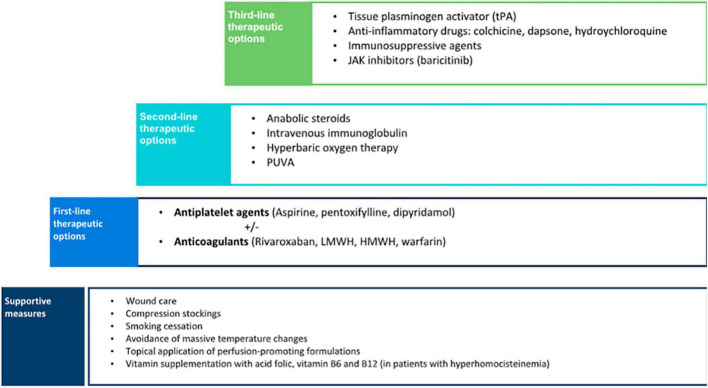
Stepped-care treatment of livedoid vasculopathy.

As LV is painful and often scarring, it is mandatory to establish a treatment. Pain management, wound care, control of cardiovascular risk factors, and anticoagulants could be the main treatment options (Figure 7).

Despite a growing number of therapies, some based on a successful case, LV treatment still represents a challenge as no single therapeutic approach is effective for all patients and there are no standardized guidelines available due to the low incidence of the disease and lack of large studies. There are several recent reviews focusing mostly on rivaroxaban and intravenous immunoglobulin (38–40).

Supportive measures are the basic step to managing patients with LV. Cessation of smoking is a crucial preventive measure as vasoconstriction and hypoxia may increase tissue damage. Despite the negative effects of smoking on wound healing, a significantly higher proportion of smokers with LV was found compared with the control population (16). Therefore, patients should be advised to enter a smoking cessation program. Compression therapy may also be beneficial, especially in patients with venous insufficiency, as it reduces edema and improves ulcer healing (10). Other preventive measures include avoidance of massive temperature changes and the topical application of perfusion-promoting formulations (18).

Although the optimal treatment approach for LV remains controversial, some authors proposed a therapeutic stair in which the first-line step is antiplatelet therapy including agents such as clopidogrel, ticlopidine, abciximab, buflomedil hydrochloride, and beraprost sodium, but most of the cases are treated with aspirin, pentoxifylline, and dipyramidole (9). These agents have been successfully used either as monotherapy or combined (38). The mechanism of action of aspirin is through the inhibition of cyclooxygenase that suppresses thromboxane A_2_ and prostaglandin I_2_, resulting in an antithrombotic effect (10). Aspirin has also proved to help ulcer healing in the treatment of chronic venous leg ulceration in previous studies (41). The recommended dose ranges from 75 to 325 mg three times a day. A significant improvement has been reported in patients with LV associated with sickle cell trait when treated with aspirin (42). Dipyramidole inhibits the synthesis of thromboxane A_2_ and stimulates the release of prostaglandin I_2_. It is usually given in a dose of 50 mg, three times a day (10). Pentoxifylline, a competitive non-selective phosphodiesterase inhibitor, has a hemorheological effect, and its recommended dose is 400 mg three times a day. The low cost, tolerability, and wide availability are important advantages of antiplatelet agents that make them a good first-line therapy option.

If there is no significant improvement in a short period after the instauration of antiplatelet treatment and in cases with a demonstrated thrombophilia, the next step in the therapeutic approach of LV is the initiation of anticoagulants (9). In this case, warfarin, heparin, low-molecular-weight heparin, other vitamin K antagonists, sulodexide, and mostly rivaroxaban have been used (9). A recent systematic review showed that anticoagulants were the most commonly reported monotherapy, achieving a consistent favorable response in up to 98% of the patients (38). Rivaroxaban was the anticoagulant most frequently used, followed by low-molecular-weight heparin, high-molecular-weight heparin, warfarin, and fluindione. The good response observed in patients with LV to anticoagulants supports the proposed pathogenic mechanism of a locally increased occlusive vasculopathy.

In the past years, rivaroxaban, a direct factor Xa inhibitor, has been widely used for the treatment and prevention of major thromboembolic diseases. Compared with other anticoagulants such as enoxaparin and warfarin, rivaroxaban is often preferred due to the advantage of oral administration and the unnecessity of international normalized ratio monitoring. In 2013, Kerk et al. first reported that patients with LV were successfully treated with rivaroxaban (43). Since then, several case reports and case series have been published treating up to 73 patients in a recent review (39). It is of note that rivaroxaban is the only drug with a clinical trial involving 25 patients with LV (RILIVA), showing statistically significant improvement in pain with a mean score of 65 to 6 on a 0- to 100-point visual analog scale after 12 weeks of treatment (6). Acute pain due to cutaneous infarction is a great challenge in LV. The results of this study showed that pain was reduced by 50% within 11 days. A recent systematic review found that a rivaroxaban dose of 10–20 mg/day was effective in 82.2% of patients with LV with thrombophilic factors as well as in those with idiopathic disease (39). Furthermore, improvement of pain can be observed soon within the first week and remission lasted 4 weeks to 23 months with an initial dose of 20 mg that can be tapered to 10 mg/day for maintenance (39). Few adverse effects were observed, with menorrhagia being the most commonly reported in the RILIVA clinical trial (6). Therefore, clinical evidence suggests that rivaroxaban is an effective and well-tolerated drug for LV.

Anabolic steroids were the second most frequently reported monotherapy for the treatment of LV (38). Danazol was the most commonly used steroid, given in a dose of 200 mg/day, although stanozolol 4 mg/day can also be used (38, 44). Systemic steroids increase fibrinolysis, inhibit coagulation, and induce hepatic synthesis of protease inhibitors (such as proteins C and S). Steroids have been reported to be an effective option, especially in patients with an associated connective tissue disease (9, 38). Despite being considered the second option, when we performed a survey in our country including dermatologist management of up to 200 patients (unpublished data), if supportive measures and anti-aggregation or hemorheological agents were not enough, the second line was mostly anticoagulants. Our experience is aligned with combination therapy including anti-inflammatory drugs with anticoagulant therapy in a Thai cohort (12).

Treatments such as intravenous immunoglobulin, psoralen and UV-A (PUVA), and hyperbaric oxygen therapy (HBOT), which have reported favorable clinical outcomes, may be more suitable for refractory cases of LV, because of high cost and difficulties in patient compliance (9, 38). Intravenous immunoglobulin was the third most commonly used and effective treatment (38). A recent review has found 3 studies and 14 case reports and series encompassing up to 80 treated patients, mostly females (70%) with 22.2% of them presenting positive thrombophilic factors and refractory to previous treatments, although rivaroxaban was not used in any of these patients (40). Although the mechanism of action is not fully understood, it induces a reduction of cytokine production, neutralization of pathogens, inhibition of complement-mediated damage, and blockage of Fc receptors (4). It is often used as monthly infusions with a recommended dose of 2 g/kg (45). Reported efficacy is high with 95% of the global response, starting between the first and third cycles, decreased up to 80% of values of the visual analog scale, and no differences between patients with thrombophilic factors and idiopathic ones (40). Treatment intervals can be prolonged based on its efficacy and it is noteworthy an improvement in dysesthesia. Remission periods are quite good, ranging from 3 months to 8 years. Although the most common adverse event is headache, intravenous immunoglobulin is an effective and safe therapy for LV, and due to the high cost and relatively complex administration, it is preferred as an alternative therapy for refractory LV cases. PUVA has been used for the treatment of LV in a small number of patients with good outcomes and minimal adverse events (46). HBOT has also been reported to be an effective alternative for LV (47, 48). HBOT releases 100% oxygen at high pressures, increasing tissue oxygenation and improving tissue ischemia. It also promotes fibrinolysis and angiogenesis, resulting in better ulcer healing (38).

There are other treatment alternatives, including fibrinolytics, vasodilators, anti-inflammatory, and immunosuppressive agents, that may be considered when conventional therapies have failed and should be reserved as the third-line option. Fibrinolysis with recombinant tissue plasminogen activator (tPA) has been reported as an effective treatment for non-healing ulcers in LV (49). It is suggested that tPA lyses microvascular thrombi, restores circulation, and eventually promotes wound healing. The recommended dose is 10 mg administered intravenously, a much smaller dose than the one used to treat other thrombotic diseases; nevertheless, the risk for severe bleeding-related adverse events still exists and the efficacy and safety of tPA should be further studied (38). The use of vasodilators such as nifedipine was found to be useful as adjuvant therapy in anecdotical reports (50). Anti-inflammatory drugs including colchicine, dapsone, and hydroxychloroquine have also shown a favorable response (51). The use of corticosteroids in the treatment of LV is controversial. Prednisolone has principally been used in flares up for rapid disease control with good results (10).

Vitamin supplementation with folic acid, vitamin B6, and vitamin B12, all being the cofactors of homocysteine metabolism, can be considered in cases of demonstrated hyperhomocysteinemia (52).

It has recently been reported the use of baricitinib, a relatively new JAK 1 and JAK 2 inhibitor, to treat 3 cases of LV that were resistant to conventional therapy showed marked improvement and no adverse events (53). Another pilot study included 5 patients refractory to danazol or corticosteroids treated with etanercept 25–50 mg once a week for 12 weeks with a pain reduction of 34.3% (54). Thus, anti-TNF and JAK inhibitors emerge as new targets and potentially effective therapeutic alternatives for LV, although further studies are needed to confirm their efficacy and long-term safety. Currently, the best evidence supporting use favors anticoagulants, especially rivaroxaban, antiplatelets, and intravenous immunoglobulin as well as supportive measures.

## Author contributions

ML-V and MS contributed to conception and design of the study, wrote the first draft of the manuscript, and prepared the graphs to illustrate it. Both authors contributed to manuscript revision, read, and approved the submitted version.
